# Endoscopic-Assisted Ommaya Reservoir Placement: Technical Note

**DOI:** 10.7759/cureus.1490

**Published:** 2017-07-19

**Authors:** Jessica Lane, Brad E Zacharia

**Affiliations:** 1 Department of Neurosurgery, Penn State Hershey Medical Center

**Keywords:** ommaya reservoir

## Abstract

Ommaya reservoir placement has been an option for patients requiring cerebrospinal fluid (CSF) access since the 1960s. It is preferred to repeat lumbar punctures, both in terms of patient comfort and the consistency of intrathecal drug concentration. Technological developments have advanced the placement technique, allowing for better accuracy and reduced complications. Freehand placement was first augmented with pneumoencephalograms and intraoperative computerized tomography (CT), then with optical-based navigation, and finally by utilizing electromagnetic neuronavigation. We outline a method of placement using electromagnetic neuronavigation and intraoperative endoscopic visualization, which allows for both real-time guidance and the confirmation of placement while maintaining tract patency for the entirety of the procedure. We make our incision and burr hole near Kocher’s point. The neuronavigation stylet is placed in a peel-away sheath (Cook Medical, Bloomington, Indiana, US), which allows us to advance into the ventricle under real-time neuronavigation guidance. After the ventricle is entered, the stylet may be withdrawn and an endoscope advanced down the sheath. The intraventricular anatomy and catheter placement are confirmed. The burr hole reservoir is attached to a ventricle catheter that has been trimmed based on trajectory measurement on preoperative imaging. The reservoir-catheter construct can then be placed and the sheath removed from around it. This method provides a high level of confidence in appropriate catheter placement.

## Introduction

The Ommaya reservoir was initially developed in 1963 and has since become an important neurosurgical tool for patients requiring repeated cerebrospinal fluid (CSF) sampling or intrathecal therapeutics [1]. While these devices can be used for a number of conditions, e.g., intraventricular hemorrhages, tumor cyst aspiration, meningitis, and neoplastic diseases, they are most commonly utilized for the instillation of intraventricular chemotherapy for the treatment of neoplastic meningitis and central nervous system (CNS) lymphoma. The placement of an Ommaya reservoir allows for the repeated outpatient administration of chemotherapeutic agents, as well as the potential for routine CSF monitoring for treatment response. Treatment and CSF sampling can be performed via repeated lumbar punctures (LPs), however, the administration of chemotherapy via a reservoir has higher and more consistent drug concentrations compared to an LP as well as less discomfort for the patient [2].

The benefits of an Ommaya reservoir, however, do not come without some degree of risk. Early series estimated that the rate of major hemorrhage was around three percent. More recent studies have demonstrated decreased rates, perhaps attributed to surgeon experience, modern operative tools and techniques, and careful analysis of preoperative coagulation parameters [3-4]. Infection remains a concern, given the immunocompromised status of many of these patients and the routine access to the reservoirs. Two recent retrospective studies demonstrated the rates of Ommaya reservoir infection at 5.5 percent and 8 percent [5-6].

Another common complication of reservoir placement is suboptimal catheter positioning. Because these devices are most often utilized for the administration of cytotoxic chemotherapeutics, optimal placement is essential. Malpositioned catheters may result in treatment delays, neurologic morbidity, and a return to the operating room for repositioning. Contrary to patients with hydrocephalus, this patient cohort often presents with normal or small ventricular size, further increasing this risk. The published rates of suboptimal ventricular catheter placements vary widely and are somewhat dependent on the stringency of the definition of accuracy. Even within groups using neuronavigation, rates of suboptimal placement range from 1 - 25 percent [3-4,7-8].

While initial methods for the placement of ventricular catheters for reservoirs or ventricular shunts relied on external landmarks, the development of imaging guidance has allowed for accurate targeting. The introduction of stereotactic placement methods, initially with frame-based systems, has afforded accurate targeting [9]. Frameless optical stereotaxy is an incremental improvement, providing visual trajectory guidance without compromising safety and accuracy [10]. Frameless, electromagnetic neuronavigation systems are the latest in technology, delivering real-time guidance during catheter placement (without the need for rigid fixation) and demonstrating improved accuracy and reduced operative time [4]. While providing accurate ventricular targeting, these techniques do not provide the real-time confirmation of optimal catheter placement. Although it can increase operative time and cause patient discomfort, the instillation of ventricular air (pneumoencephalogram) allows for the confirmation of appropriate catheter tip placement before leaving the operating theater. Finally, an intraoperative CT scan allows for the confirmation of placement but is not available in all centers.

Given the myriad available technologies, no single methodology has become the gold standard. Here, we present a technical modification of Ommaya reservoir placement utilizing frameless, electromagnetic neuronavigation and ventricular endoscopy, providing guidance into the ventricular system and the real-time confirmation of accurate placement.

## Technical report

General Surgical Considerations

After the standard considerations of Ommaya reservoir placement, patients undergo either a preoperative high-resolution magnetic resonance imaging (MRI) or CT scan of the brain for surgical planning. We routinely hold anticoagulation and antiplatelet agents for approximately one week pre- and postoperatively. This patient population often has some degree of myelosuppression, and particular attention is paid to preoperative complete blood count (CBC) and coagulation studies. It is our practice to aim for a platelet count of more than 100,000 and an international normalized ratio (INR) of less than 1.4. If the procedure is needed urgently, we will transfuse platelets and/or fresh frozen plasma (FFP) or prothrombin clotting complex (PCC) preoperatively to obtain these levels. In patients receiving systemic chemotherapy, we work with their oncologists to time the procedure to avoid the nadir of their myelosuppression. We typically observe patients overnight, and their first intrathecal treatment is administered the next morning, prior to discharge. We do not routinely obtain confirmatory postoperative cranial imaging prior to the use of the device. 

Reservoir Placement

**Figure 1:**
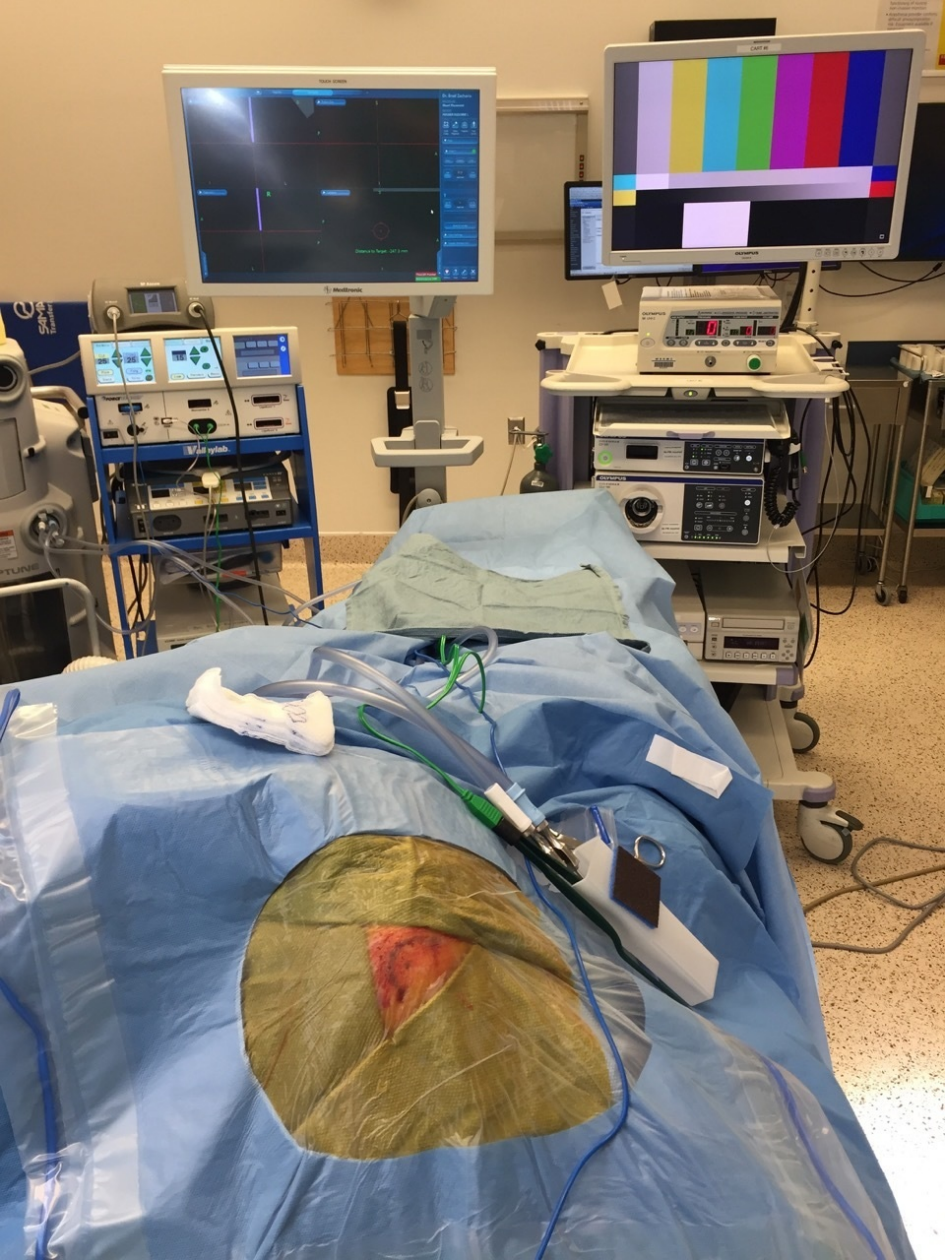
Set-Up Draped set-up with both endoscope and neuronavigation screens

The patient is positioned supine on a standard operating table (Maquet Medical Systems, Rastatt, Germany). The patient is then turned 90 degrees from the direction of administration of anesthesia, with the patient’s right side facing the surgeon for a planned right frontal approach. While a right-sided approach is our standard operating procedure (SOP), we will perform the procedure on the left if anatomy or pathology so dictate. Screens for both the neuronavigation and the endoscope system are placed at the foot of the bed for easy visualization during catheter advancement (Figure 1).

**Figure 2:**
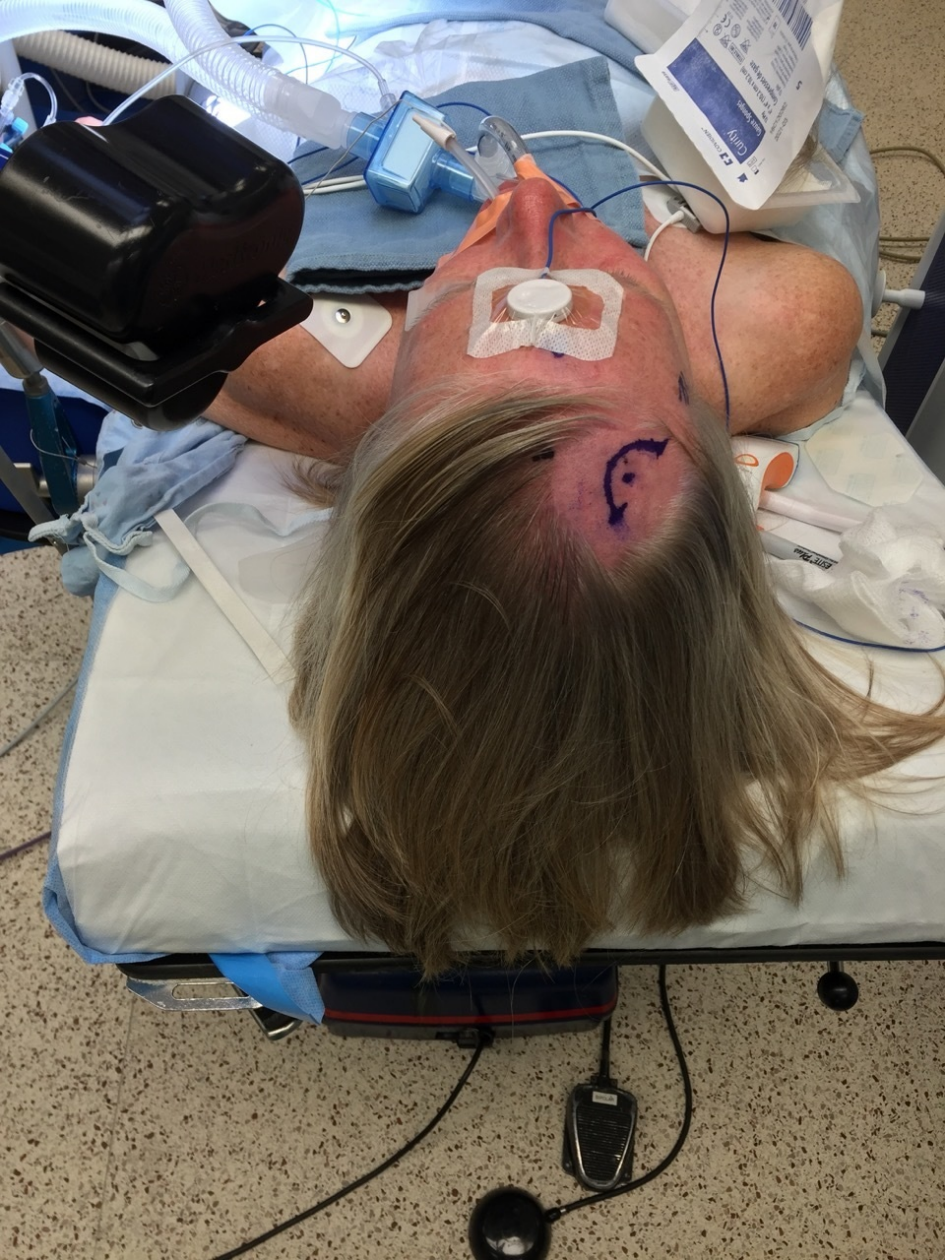
Planned Curvilinear Incision, Laterally Oriented Axiem reference array placed on the patient's forehead with mastisol and tegaderm; Axiem transmitter contralateral to reservoir placement, approximately a hand width from the temple

Using the StealthStation S7 AxiEM navigation system, (Medtronic, Inc., Dublin, Ireland), we place the reference array in the mid-forehead. We utilize Mastisol (Eloquest Healthcare, Inc., Ferndale, MI) to improve adhesion and cover the array with a small Tegaderm dressing (3M, St. Paul, MN) (Figure 2).

The system is registered to the patient using the AxiEM registration probe (Medtronic, Dublin, Republic of Ireland). The accuracy of the neuronavigation system is confirmed using multiple surface landmarks. Stealth trajectory planning software is then utilized to select a target just superior to the right foramen of Monro. At this stage, we identify Kocher’s point via anatomic landmarks and measurements. We then place the probe at this point and navigate to the entry point. This trajectory is reviewed and confirmed on the stealth system to traverse a gyrus and avoid sulcal boundaries and cortical vessels. To achieve this goal, small adjustments can be made to the entry and target. After a final entry point has been selected, a curvilinear incision is made to allow for the reservoir to be placed under intact scalp tissue. The entry is angled laterally in the event that the reservoir requires augmentation to a ventriculoperitoneal shunt in the future (an event which occurs in roughly 15 percent of patients).

The region is prepped and draped in the standard sterile fashion. A local anesthetic is administered with epinephrine for hemostasis and pain control. The skin and scalp are incised with a #15 blade. Dissection is carried down to the bone using monopolar electrocautery and the periosteum is elevated. A 2-0 vicryl suture is used to hold the skin flap back, to avoid interference with the navigation caused by a metallic retractor. A single burr hole is placed at the previously identified entry point with a standard 14/11 mm perforator (ACRA-CUT, Inc., Acton, MA). Hemostasis is obtained and the dura is cauterized.

**Figure 3:**
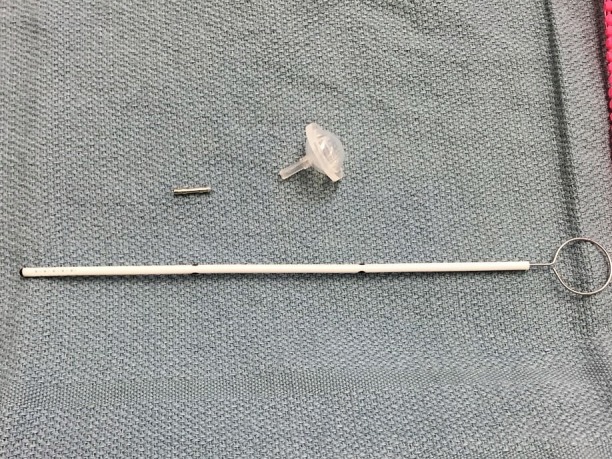
Ommaya Reservoir Ommaya reservoir, metal connector pin, and untrimmed peel-away sheath

Next, the Ommaya reservoir is prepped. The distance from the outer table of the skull to the target is determined via the preoperatively planned trajectory. The ventricular catheter is cut 1.5 cm shorter than this distance to account for the additional length added from the reservoir and metal connector. This cut ventricular catheter is attached to a 14 mm burr-hole-style reservoir (Codman, Inc., Raynham, MA) with a single metal connector. The catheter is secured with two 2-0 silk ties (Figure 3).

**Figure 4:**
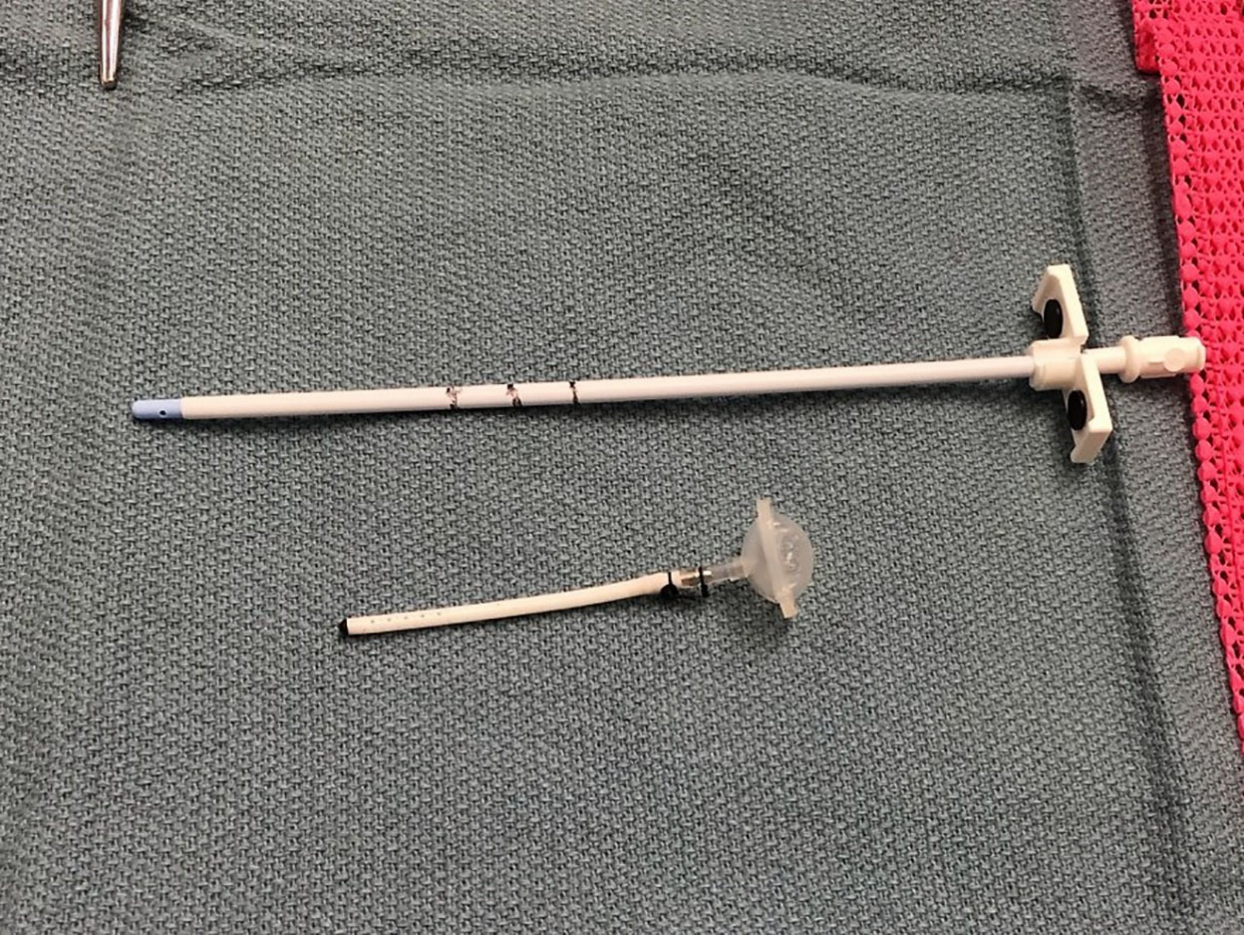
Ommaya Reservoir Reservoir secured to a trimmed ventricular catheter with silk ties and a marked peel-away introducer sheath

The reservoir is not flushed prior to insertion to provide a clear visualization of the CSF in the reservoir after appropriate placement. We then prepare the 9 French peel-away introducer sheath by placing 5, 6, 7, and 8 cm markings on the outer sleeve of the sheath (Figure 4). 

A Minop 0 degree ventriculoscope (Aesculap, Inc., Tuttlingen, Germany), without the obturator, is attached to the camera head and appropriately oriented, focused, and white balanced.

Next, the dura is sharply opened in a cruciate fashion with a #11 blade and the leaflets are cauterized. The pia and arachnoid are cauterized and sharply opened to allow for the slightly larger size of the introducer sheath. The AxiEM navigation stylet is then placed into the sheath. The sheath-stylet complex is then positioned at the planned point of entry on a gyral surface and the planned trajectory to the foramen of Monroe is verified on the neuronavigation system. The sheath-stylet construct is passed toward the target using the neuronavigation guidance and trajectories views, providing real-time tracking of the position of the stylet tip relative to the target. The sheath-stylet construct is advanced just to the point of ventricular entry, at which point the stylet and obturator are removed, leaving the sheath behind. Care should be taken to enter only the ventricle while avoiding the choroid plexus, which could cause minor bleeding and obscure visualization.

The endoscope is then passed through the sheath and relevant ventricular anatomy, including the foramen of Monroe, and the septal and thalamostriate veins are visualized. If minor bleeding occurs at this point, copious irrigation generally clears it. Although we have not encountered it, should substantial bleeding occur, obscuring clear visualization despite irrigation, we recommend aborting the procedure and leaving an external ventricular drain in place. After the foramen is visualized, the scope is gently advanced to or through the foramen of Monro and the sheath is advanced over the scope to the correct depth. This is further confirmed with the external markings on the sheath. The scope is then removed and the sheath is peeled down to the surface of the skull.

The unitized Ommaya reservoir and catheter are then advanced down the sheath. As the metal connector is reached, there will be some mild resistance. With the surgeon holding the metal connector securely, the assistant gently peels the sheath to completion and the reservoir is seated. The Ommaya reservoir is pumped to ensure the adequate filling of clear CSF. The wound is copiously irrigated and meticulous hemostasis is obtained. The galea is closed with 3-0 vicryl sutures and the skin with 3-0 running monocryl. Finally, the wound is cleansed and dressed simply with bacitracin ointment. 

## Discussion

We present a technical report demonstrating the optimal placement of the Ommaya reservoir using both electromagnetic neuronavigation and ventricular endoscopy. While a variety of tools and techniques have been previously utilized, this technique offers several distinct advantages. We feel that neuronavigation is particularly useful for Ommaya reservoir placement, given the propensity for patients to have normal to small ventricular size. Neuronavigation assists in ventricular access and can provide an ideal planned trajectory toward the foramen of Monro while avoiding sulcal boundaries, theoretically reducing the risk of intracranial hemorrhage. Electromagnetic neuronavigation has a number of advantages over the frequently used optical-based navigation. First, there is no requirement for rigid fixation, which may minimize anesthetic requirements. In addition, the stylet is navigated at the tip, perhaps providing more accurate navigation by avoiding line-of-sight issues. Utilizing this technology alone, one may successfully place the majority of proximal catheters; however, no real-time confirmation is provided. Utilizing our method, real-time confirmation is provided using endoscopy. Formal ventricular endoscopy provides a panoramic view of ventricular anatomy, confirming the location. The endoscope is passed through the sheath for visualization and then removed allowing for the placement of the unitized Ommaya catheter without loss of tract patency through the procedure.

## Conclusions

This report outlines a method of Ommaya reservoir placement that addresses a number of risks with previous techniques. As a technical report, it lacks a direct comparison of precision or complication rate with alternative techniques. We perform a high-volume of Ommaya reservoir placements. When utilizing this technique, we have found a high degree of accuracy coupled with a low complication rate. In our experience, no catheters have required repositioning and we have successfully cannulated all patients. We cannot, however, claim this technique to be superior to the numerous others described in the literature. Future reports may describe, more quantitatively, our success with this technique and compare it to published cohorts. We hope that this technical note adds to the surgical armamentarium for the placement of the Ommaya reservoir and the proximal ventricular catheter. 
